# Successful Cryopreservation of Dormant Buds of Blackcurrant (*Ribes nigrum* L.) by Using Greenhouse-Grown Plants and In Vitro Recovery

**DOI:** 10.3390/plants10071414

**Published:** 2021-07-10

**Authors:** Saija Rantala, Janne Kaseva, Anna Nukari, Jaana Laamanen, Merja Veteläinen, Hely Häggman, Saila Karhu

**Affiliations:** 1 Natural Resources Institute Finland (Luke), Production Systems, Survontie 9 A, FI-40500 Jyväskylä, Finland; 2 Ecology and Genetics Unit, Faculty of Science, University of Oulu, P.O. Box 3000, FI-90014 Oulu, Finland; hely.haggman@oulu.fi; 3 Natural Resources Institute Finland (Luke), Natural Resources, Tietotie 4, FI-31600 Jokioinen, Finland; janne.kaseva@luke.fi; 4 Natural Resources Institute Finland (Luke), Production Systems, Latokartanonkaari 9, FI-00790 Helsinki, Finland; anna.nukari@luke.fi; 5 Natural Resources Institute Finland (Luke), Natural Resources, Survontie 9 A, FI-40500 Jyväskylä, Finland; jaana.laamanen@luke.fi; 6 Boreal Plant Breeding Ltd., Myllytie 10, FI-31600 Jokioinen, Finland; merja.vetelainen@boreal.fi; 7 Natural Resources Institute Finland (Luke), Production Systems, Itäinen Pitkäkatu 4 A, FI-20520 Turku, Finland; saila.karhu@luke.fi

**Keywords:** currants, cryobanking, plant genebanks, plant genetic resources, germplasm collections, liquid nitrogen, long-term preservation, safety back-ups

## Abstract

The cryopreservation of dormant buds can be a feasible method for preserving germplasm of cold-tolerant woody plants. In the present study, we evaluated the effects of pre-desiccation, thawing method, and the rehydration of bud sections on the post-cryopreservation recovery of dormant blackcurrant buds in vitro. The estimated recovery of small- and medium-sized buds was 80.1 and 62.7% respectively for desiccated buds and 67.8 and 72.3% respectively for non-desiccated buds. The pre-desiccation of bud sections enhanced the number of the shoots regenerated from vegetative buds (2.3 vs. 4.7). The estimated recovery of fast-thawed buds was better after 14-day than after 7-day rehydration (85 vs. 59%). In slowly thawed buds the difference between 14-day and 7-day rehydration was not significant (73 vs. 62%). The estimated recovery of vegetative and flower buds was 77.7 and 41.1% respectively after 7-day rehydration, and 95.2 and 43.6% respectively after a 14-day rehydration period. The rehydration of bud sections was not necessary for the in vitro recovery of non-desiccated, fast-thawed buds. Of the 23 blackcurrant cultivars cryopreserved using non-desiccated dormant buds collected from a greenhouse, the estimated recovery of 22 cultivars ranged between 42 and 90%.

## 1. Introduction

The preservation of the genetic resources of agricultural crop plants is important for future plant breeding and food security. The germplasm of vegetatively propagated crops can be maintained as plants in the field or in greenhouses or as in vitro cultures [1,2]. The maintenance of collections solely in the field is a risk, due to the fact that diseases, pests, and adverse weather conditions can compromise the preservation of germplasm. A duplicate field collection or a safety backup by an alternative conservation method is therefore recommended by FAO [1]. Cryopreservation, i.e., the preservation of biological material at ultralow temperatures (in liquid nitrogen and/or its vapor phase at temperatures ca. −196 °C to −140 °C [3]), is a useful and cost-effective option to secure the long-term preservation of plant germplasm [4,5,6]. For example, in the case of clonally propagated fruit trees that are traditionally maintained in clonal orchards, the utilization of cryopreservation can greatly improve the conservation of germplasm [7]. However, to utilize cryogenic preservation, cryoprotocols suitable for the species or even genotypes in question are needed.

For the long-term preservation of cold-tolerant woody plant species, cryopreservation of dormant buds may be an applicable method [8,9]. The success of dormant bud cryopreservation is affected by the quality of the source material, the steps of the protocol, and recovery practices [8]. Cryopreservation of dormant buds was reported in the 1960s when Sakai discovered that the twigs of cold-hardy poplar (*Populus sieboldii* Mig.) and willow (*Salix koriyanagi* Kimura) pre-frozen at −30 °C were able to survive without fatal intracellular freezing even when immersed in liquid nitrogen [10]. After that, the cryopreservation of dormant buds was studied with species such as apple (*Malus domestica*) [11,12] and mulberry (*Morus bombycis* Koidz.) [13], and to date, many cryoprotocols utilizing dormant buds with different recovery practices have been developed [8,9]. In the case of *Malus*, the recovery of buds is often done by grafting [14], but the recovery of buds via in vitro can also be used as in the case of species such as *Ulmus* [15], *Diospyros kaki* Thunb. [16], *Morus* [13,17], *Betula pendula* Roth, and *Populus tremula* L × *P. tremuloides* Michx. [18]. The cryopreserved twigs of *Salix* can be recovered via direct rooting by placing thawed twigs in moist, sterilized soil and keeping them in high humidity until they have rooted [19,20]. In some temperate fruit trees, thawed twig segments can be forced, after which sprouted shoots can be excised and introduced into the tissue culture [21]. Shoot tips excised from dormant buds prior to cryopreservation may also be utilized for cryopreservation with cryoprotectants [22]. The success of dormant bud cryopreservation varies depending on the protocol and species, e.g., for *Malus* spp., recovery ranges of accessions from 16 to 100% was reported [23].

The pre-desiccation of bud sections has been proven to enhance the post-cryopreservation recovery of dormant buds [11,12,24] by decreasing cells’ water content and preventing intracellular ice crystallization of cryopreserved material during cooling and thawing [25]. Therefore, many dormant bud cryoprotocols include the pre-desiccation of bud sections, e.g., in the protocol developed for *Malus* species [23], bud sections are desiccated to a moisture content of 25–30% before slow cooling to a range between −30 and −40 °C, followed by a transfer to liquid nitrogen or its vapor phase. This protocol is used or slightly modified also for some species, e.g., for *Fraxinus* [26] and *Vaccinium* [27].

However, careful monitoring is often needed for evaluating the progress of desiccation, which is usually laborious and needs time. Cryopreservation of non-desiccated dormant buds recovered via sprouting or grafting was reported for *Salix* [28] and for *Malus* [29] but the pre-desiccation of buds is usually omitted from protocols in which the recovery of buds is achieved via in vitro culture, as in the case of *Ulmus* [15] and *Betula pendula* Roth [18].

Blackcurrant *Ribes nigrum* L. is a cold-tolerant woody shrub, and it is cultivated for juicy berries both commercially and in home gardens. According to the FAOSTAT database, the production of currants (mainly blackcurrant) was 647,815 tonnes in 2019 [30]. Many old blackcurrant varieties or local strains are no longer used in commercial berry production, but they may be valuable source for future plant breeding. However, plants preserved in the open field are often exposed to many pests and diseases. Pests such as eriophyid mites, spider mites, moths, gall midges, and aphids are common in currant cultivation in Finland. Fungal diseases, pathogens causing leaf spots, powdery mildew, and rusts may also occur, but these do not always cause severe problems in blackcurrants in Finland [31]. Certain pests may also act as vectors for viruses, and several virus diseases may occur in blackcurrants [32]. Blackcurrant reversion virus (BRV), transmitted by the gall mite (*Cecidophyopsis ribis*), is the most significant virus in blackcurrants, causing disease symptoms to the leaves and flowers, proliferation, and ultimately yield losses [33,34]. Pest and disease infections can be prevented in certified plant production by maintaining pre-basic mother plants (stock material) in insect-proof greenhouse [35].

The Finnish national core collection of blackcurrant includes a total of 27 cultivars and landraces, all called cultivars in this study. The core collection was selected as part of the multinational RIBESCO project in 2007–2011 [36,37]. It is managed by the Finnish National Genetic Resources Programme for Agriculture, Forestry, and Fishery and maintained by the Natural Resources Institute Finland (Luke). A new field collection of the blackcurrant genebank was established in Kaarina, Finland between 2011 and 2016 because of the symptoms of blackcurrant reversion virus were detected in the old field collection. The renewal of field collection was conducted using plants produced via micropropagation. In the context of the renewal of new field collection, the need to create a cryopreserved backup collection was identified. Blackcurrant can be cryopreserved by using explants excised from tissue cultures [38,39,40,41] or by using dormant buds [42,43,44]. Cryopreservation success of blackcurrant varies depending on cultivar. According to our previous study, the estimated post-cryopreservation recovery of in vitro-derived shoot tips ranged by cultivars from 17 to 94% [41]. Previously, we also studied the cryopreservation of dormant blackcurrant buds using greenhouse and field-maintained source plants of the cultivar Mortti, and we reported the estimated post-cryopreservation recovery of buds in vitro from 66 to 86% [43]. In eleven blackcurrant cultivars, a post-cryopreservation viability of dormant buds from 58.9 ± 1.1% to 73.5 ± 1.9% in field conditions was reported [44].

The aim of the present study was to evaluate the suitability of dormant bud cryopreservation for the preservation of the blackcurrant germplasm collection using dormant buds derived from greenhouse-maintained plants. Dormant buds of cvs. Mikael, Marski, and Vilma were cryopreserved according to an experiment setup to confirm the utility of a selected cryoprotocol and to evaluate the necessity of pre-desiccation and rehydration of bud sections, and the effect of the thawing method (fast or slow) on the in vitro recovery of cryopreserved buds. Finally, the post-cryopreservation viability of non-desiccated, fast-thawed dormant buds of 24 cultivars was estimated.

## 2. Results

### 2.1. Protocol Experiments

The effect of desiccation, thawing method, and rehydration of bud sections was tested according to the experimental set-up with cvs. Mikael, Marski, and Vilma. The actual recovery percentages of cryopreserved buds varied from 20 to 80%, depending on the cultivar and treatment combination (Table 1). The estimated recovery of cryopreserved buds over all treatment combinations was 60, 83, and 67% for cvs. Mikael, Marski, and Vilma, respectively. Cryopreserved buds were mainly floral; out of 160 buds per cultivar, the numbers of vegetative buds were 21, 37, and 52 for cvs. Mikael, Marski, and Vilma, respectively. The average moisture content of non-desiccated twig samples ranged at 55–57%, 54–59%, and 53–55%, whereas the average moisture content of desiccated twig samples ranged at 22–32%, 31–34%, and 30–33% for cvs. Mikael, Marski, and Vilma, respectively. In all three cultivars, the recovery of non-cryopreserved control buds ranged from 90 to 100% for both non-desiccated buds and buds that were desiccated and thereafter rehydrated.

#### 2.1.1. Preliminary Quality Evaluation

When the in vitro cultures were initiated from cryopreserved buds, the percentages of healthy green buds, i.e., buds without blackening or paleness of shoot tip, flower primordia, or leaves, were 71, 68, and 36 for cvs. Mikael, Marski, and Vilma, respectively. According to the results of all three cultivars, the percentages of healthy-looking green buds were higher (i) if buds were desiccated as opposed to when they were not (70 vs. 48%; *p* < 0.001), (ii) if buds were thawed slowly instead of fast thawing (76 vs. 40%; *p* < 0.001), (iii) if buds were small instead of medium-sized buds (67 vs. 54%; *p* = 0.011), and iv) if buds were rehydrated for 7 days instead of 14 days (63 vs. 54%; *p* = 0.052). The frequency of buds without visual damage did not differ between flower buds (58%) and vegetative buds (60%). However, almost all buds (28 out of 30) with their outermost leaves blackened were flower buds of cv. Marski. Leaves with no turgor (i.e., not fully rehydrated) occurred only in buds that were desiccated and then rehydrated after thawing for 7 days. Of 40 buds per cultivar desiccated and rehydrated for only 7 days, the number of not fully rehydrated buds was 18 for cv. Mikael and was 36 for cvs. Marski and Vilma.

Notably, the presence of these visual damage of buds did not influence the later recovery percentage of the in vitro cultures (*p* = 0.576). Of the 244 buds that recovered in vitro, 105 (43%) had visual symptoms of damage when the in vitro cultures were initiated. Furthermore, of the 228 buds that did not recover, 136 (60%) did not have visual symptoms of damage at the time of initiation of in vitro cultures.

#### 2.1.2. The Effect of Desiccation on the Recovery of Thawed Buds In Vitro

**Figure 1:**
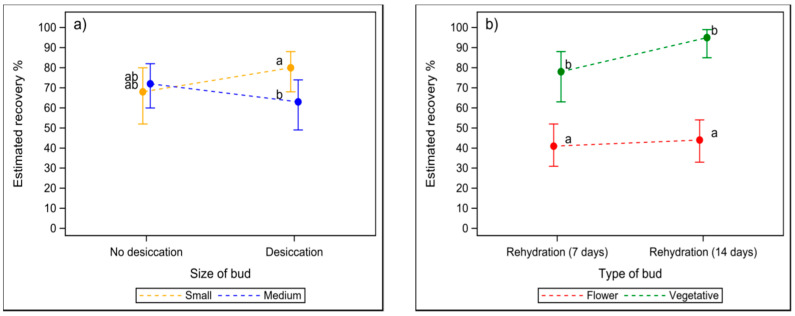
The estimated mean recovery of cryopreserved buds of blackcurrant cvs. Marski, Mikael, and Vilma according to (**a**) the pre-treatment of bud sections (desiccated or not) and the bud length, and (**b**) the bud type and rehydration time of bud sections. The effects of cultivar, pre-treatment, thawing method, rehydration, type of bud, size of bud, and all their 2-way and 3-way interactions were analysed using generalised linear mixed models. The length of the bars indicates the sizes of 95% confidence intervals. Letters a and b indicate significant differences (*p* < 0.05) in estimated recovery rate in vitro between treatments.

According to the statistical analysis of the data of the protocol experiment (n = 472 buds), the desiccation of bud sections of the cvs. Mikael, Marski, and Vilma had no significant main effect on the estimated recovery percentage of cryopreserved buds. An interaction was found between the pre-treatment of bud sections (desiccated or not) and the size of the buds (*p* = 0.022) (Figure 1a): desiccation improved the estimated recovery percentage of small buds slightly, but for medium-sized buds the effect was the opposite. However, the difference between desiccated and non-desiccated buds was not significant in either case. In the case of desiccated buds, the estimated recovery was better for small than for medium-sized buds (*p* = 0.037).

The number of proliferated shoots per regenerated bud evaluated 7 weeks after the initiation of in vitro cultures showed an interaction between the desiccation of bud sections (desiccated or not) and bud type (flower or vegetative) (*p* < 0.001). The estimated shoot number per bud was higher for desiccated vegetative buds than for non-desiccated vegetative buds (4.7 vs. 2.3; *p* < 0.001). In the case of flower buds, the difference between desiccated and non-desiccated buds was not significant (2.2 vs. 1.7; *p* = 0.121). In addition, the estimated shoot number per bud was higher for desiccated vegetative buds than for desiccated flower buds (4.7 vs. 2.2; *p* < 0.001), and evidence for the difference of means was found for non-desiccated vegetative and flower buds (2.3 vs. 1.7; *p* = 0.094).

The estimated number of shoots was higher for desiccated than for non-desiccated buds for cvs. Mikael (3.3. vs. 1.7; *p* < 0.001) and Marski, (3.8 vs. 2.0; *p* < 0.001) respectively, but in cv. Vilma the difference, although parallel, was not found to be statistically significant (3.1 vs. 2.3; *p* = 0.092). The difference due to bud size was rather small: the estimated number of shoots per regenerated bud was 2.9 for small buds and 2.5 for medium-sized buds (*p* = 0.051).

#### 2.1.3. The Effect of Thawing Speed and Rehydration on the Recovery of Buds In Vitro

According to the results of the protocol experiment, thawing speed had no main effect on the estimated recovery percentage of buds of cvs. Mikael, Marski, and Vilma. However, the interaction between thawing method (slow or fast) and the rehydration time of bud sections was close to significant (*p* = 0.053). The estimated recovery percentage of fast-thawed buds was better after 14-day than after 7-day rehydration (85 vs. 59%; *p* = 0.013), but in slowly thawed buds, there was no significant difference between 14-day rehydration and 7-day rehydration (73 vs. 62%; *p* = 0.637). Vegetative buds had a better estimated recovery than flower buds after both rehydration periods (*p* < 0.001). An interaction between the duration of rehydration treatment and bud type was found (*p* = 0.031). Within the bud type, the estimated recovery of buds did not differ between 7-day and 14-day rehydration treatment, although a longer rehydration time seemed to give some benefit to vegetative buds (Figure 1b). Moreover, an interaction was found between rehydration time and cultivar (*p* = 0.027). In all three cultivars, the estimated recovery of buds was better after 14-day than 7-day rehydration, but the difference was significant only for cv. Marski (92 vs. 68%; *p* = 0.016).

In cvs. Mikael, Marski, and Vilma, the thawing method and the duration of the rehydration treatment did not have significant main effects on the shoot number of regenerated buds. However, an interaction between the rehydration time and cultivar showed evidence of difference (*p* = 0.094), but in pairwise comparisons statistically significant differences could not be found.

#### 2.1.4. The Necessity of Rehydration

**Figure 2:**
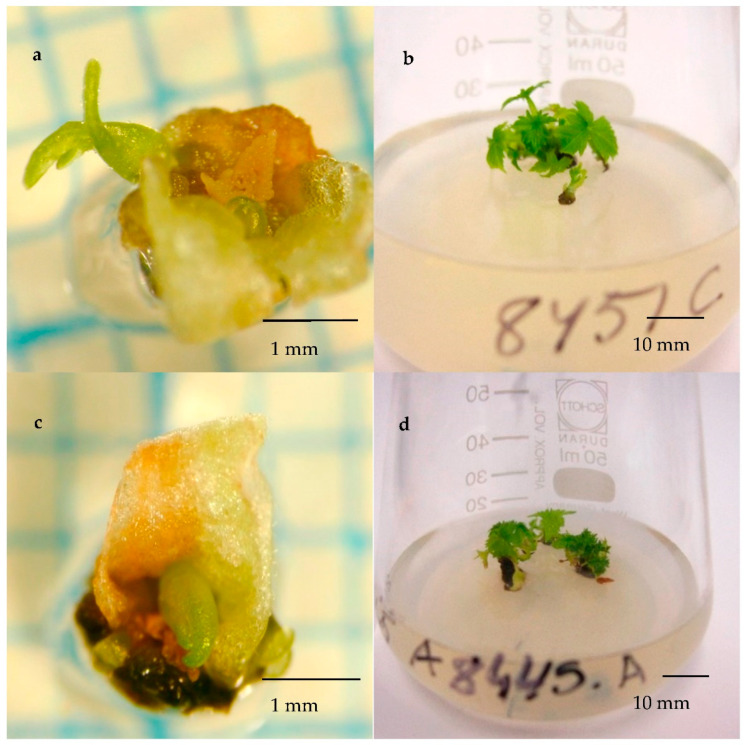
The recovery of cryopreserved buds of cv. Brödtorp in vitro. Top row: the propagule excised from a non-rehydrated bud (**a**) 2 weeks after initiation of in vitro culture (photo Dr. Mauritz Vestberg) and (**b**) 10 weeks after initiation. Bottom row: the propagule excised from a bud rehydrated 11 days (**c**) 2 weeks after initiation of in vitro culture (photo Dr. Mauritz Vestberg) and (**d**) 10 weeks after initiation. Bud sections of cv. Brödtorp were cryopreserved without pre-desiccation and revived from cryostorage via fast thawing. Images a and c were taken on graph paper through a stereomicroscope.

To evaluate the necessity of rehydration for non-desiccated, fast-thawed cryopreserved buds, 84 bud sections of cv. Brödtorp were thawed for rehydration test. The average moisture content of bud sections of cv. Brödtorp was 54%, and an average bud length was 4.5 mm (ranged from 2 to 6 mm). Ten weeks after the initiation of the in vitro culture, the estimated recovery percentage did not differ significantly between rehydrated (71 (50–86)) and non-rehydrated buds (90 (70–97), *p* = 0.087). At the time of initiation of in vitro cultures, all non-rehydrated buds were scored as “healthy green”, but only 7 of 42 rehydrated ones were “healthy green”. However, after two weeks of in vitro culture, blackening and paleness was also observed in shoot tips excised from non-rehydrated buds. In both rehydrated and non-rehydrated treatments, the first shoots started to grow two weeks after the excision of shoot tips (Figure 2). The estimated recovery of buds was again better for vegetative buds than for flower buds (94 vs. 56%; *p* = 0.003), but because of the low number of flower buds (only 6 of 84) and non-recovered buds, the test result may not be accurate.

When the shoot number of the non-desiccated, fast-thawed buds of cv. Brödtorp were analysed, no significant differences in the pairwise comparisons between rehydrated and non-rehydrated treatments could be found. However, a significant interaction between bud length (2–3 mm or 4–6 mm) and rehydration (rehydrated or not) was found (*p* = 0.047). In small buds, the estimated number of shoots was higher for rehydrated buds (5.6) compared to that of the non-rehydrated ones (2.5). The estimated shoot number for medium-sized buds did not differ between rehydrated buds (3.5) and non-rehydrated buds (3.3).

### 2.2. Viability Testing When Cryobanking a Collection of Cultivars

The results from viability assessments of 23 cultivars cryopreserved for long-term cryopreservation are shown in Table 2. All buds were cryopreserved without pre-desiccation and recovered via fast thawing and without rehydration. The estimated recovery of buds otherwise ranged from 42 to 90%, but the estimated recovery of one exceptional cv., Jänkisjärvi, was only 9%, and the difference between cultivars was not found statistically significant (*p* = 0.189). Despite a non-significant p-value of the F-test, cv. Jänkisjärvi obviously differed statistically significantly from a few cultivars. Other statistically significant differences between the estimated recovery rate of cultivars were not found because of relatively wide confidence limits.

The estimated recovery of cryopreserved buds was again better for vegetative buds than for floral bud (83 vs. 43%; *p* < 0.001). Of all the buds thawed for viability assessments, the percentage of floral buds was 6% for young donor plants and 28% for donor plants maintained in a greenhouse for several years (i.e., pre-basic mother plants or older gene bank plants). The number of flower buds varied between cultivars, with Vertti (15) having the highest number, followed by cvs. Venny (12), Jänkisjärvi (8), Mikael (6), and Hedda (6). The length of the thawed buds varied from 1–2 mm to 7 mm. Only 3 buds out of 462 were rejected due to contamination.

The estimated number of proliferated shoots per regenerated bud varied by cultivar (*p* < 0.001) and ranged between 1.1 (Pyhtilän Musta) and 5.5 (Karila) (Table 1). The estimated number of shoots per bud was higher for vegetative buds than for flower buds (3.2 vs. 2.0; *p* = 0.002).

## 3. Discussion

In the present study, dormant buds of blackcurrant were cryopreserved using a two-step freezing method. The success of cryopreservation was evaluated by in vitro recovery and the shoot formation of thawed buds.

Desiccation of buds prior to cooling is considered an essential step for the successful recovery of buds in many dormant bud cryoprotocols [45]. In the present study, the results of the protocol experiment indicated that the pre-desiccation of blackcurrant bud sections was not necessary for the post-thaw recovery of buds via in vitro culture. Results from viability assessments of a genebank collection supported this conclusion: the estimated recovery of 22 blackcurrant cultivars out of 23 that were cryopreserved without desiccation had a success of more than 40%. The result is consistent with our previous study [43] with cryopreserved blackcurrant cv. Mortti, for which the estimated recovery for non-desiccated outdoor and greenhouse-collected buds in vitro was 86 and 66%, respectively. In a previous study, the recovery of the winter buds of nine blackcurrant cultivars rehydrated 7 days before plunging into liquid nitrogen was successful via in vitro but not by grafting [42]. However, recovery by budding was reported to be successful for blackcurrant cuttings desiccated with a moisture content of 28–32% at −4 °C prior to the two-step cryopreservation [44].

According to the results of our protocol experiment, the pre-desiccation of bud sections decreased the number of buds with visual damage, but blackening and paleness were also detected in the desiccated buds. It is possible that the duration and conditions of the desiccation process were not optimal for cryopreservation, and the full benefit of pre-desiccation of bud sections was therefore not realised. It might also explain why desiccation was more effective for small buds than for medium-sized buds. The bud sections of cvs. Mikael, Marski, and Vilma were desiccated for four days at 2 °C, which is quite a short desiccation time compared to that in some other studies. For example, desiccation of 3.5 cm long stem segments of apple *Malus domestica* at −4 °C to a water content of ca. 30% of fresh weight took 11 to 14 days [46], and desiccation of 7 to 10 cm long apple sections to 28–32% moisture took 4 to 6 weeks [23]. However, dormant buds of persimmon (*Diospyros kaki* Thunb.) were desiccated at room temperature for 3 h before stepwise freezing to –30 °C in five days followed preservation at −150 °C [16]. In the case of *Diospyros kaki,* the recovery of buds was successful via in vitro but not by grafting.

The effect of thawing method on the recovery of cryopreserved buds has been studied previously with both grafted and in vitro recovered buds. The in vitro recovery of dormant buds of *Diospyros kaki*, which were dehydrated at 25 ° C for 3 h before slow cooling and cryopreservation, was better after thawing in the air at 25 °C for 24 h than after thawing at –1 °C or after thawing at 40 °C in a water bath for 15 min, plus holding at 25 °C for 24 h [16]. In vegetative buds of *Morus bombycis* Koidz., the survival rate of cryopreserved buds in vitro depended on both pre-freezing and thawing temperatures [13] When segments were slowly pre-frozen to –10 °C, rapid thawing at 37 °C for 5 min in water gave good survival rates, but slow thawing at 0 °C for at least 3 h in the air did not. When shoot segments were slowly frozen to –20 °C or –30 °C, the survival of meristems was almost 100%, regardless of the thawing method. However, the shoot formation percentage was about half that of survival, and for segments that were cooled to –30 °C, slow warming gave a better result [13]. In *Malus domestica*, the rapid warming by placing the tubes in a water bath at 30 °C for 3 min did not support the bud burst of grafted buds [47]. In the present study, the thawing method, either fast in a water bath or slowly in a cold room, did not have a statistically significant effect on the recovery of buds in vitro. We therefore concluded that the cryovials containing blackcurrant sections can be thawed in a water bath according to the protocol that was also used for *Betula pendula* and *Populus tremula* L × *P. tremuloides* Michx [18].

The results of cvs. Mikael, Marski, and Vilma indicated that the duration of the rehydration treatment (7 day or 14 day) was not significant for the recovery of the buds, although a 14-day rehydration seemed beneficial for fast-thawed vegetative buds. In addition, according to the results of cv. Brödtorp, the rehydration treatment was not necessary for non-desiccated buds. The rehydration of bud sections in moist cotton prior to the initiation of in vitro culture increases the risk of contaminations. The rehydration of bud sections was therefore not adopted, although it enhanced the regeneration of shoots.

In the present study, the bud type, i.e., floral or vegetative, had a strong effect on the success of recovery, with vegetative buds giving a better result than floral buds. For dormant bud cryopreservation, twigs from the previous season’s growth with vegetative buds are usually recommended [9,14]. Moreover, the cold hardiness and cold acclimation state of source plants is considered the most important issue affecting the success of dormant bud cryopreservation [8]. In the present study, dormant buds collected from insect-proof greenhouse-maintained donor plants were used because of their known health status and because these plants had a lower contamination risk compared to outdoor plants in the initiation of in vitro culture [48,49,50]. The exchange of the vegetative material includes the great risk of disease transfer [7]. The good health status of the source plants is beneficial for the future utilization of the cryopreserved germplasm. Cryopreserved buds of certified pre-basic mother plants may easily be used even for healthy plant production as well as for replacing old field collections after years, without new pest and disease indexing. Bud material that is examined to be free of black currant reversion virus can also be utilized later without renewing the testing of the virus.

The pre-basic mother plants for certified plant production were pruned annually, and the twigs collected for cryopreservation were from the previous season’s growth, but the prevalence of floral buds was still quite high compared to young plants. In blackcurrant, floral buds are also formed in young shoots after the first summer [51]. Both vegetative and floral buds of greenhouse-grown blackcurrant plants were used for cryopreservation because the type of intact bud is difficult to define.

The number of flower buds among cryopreserved buds still in a cryotank cannot be known, but the possibility that a proportion of the cryopreserved buds would be flower buds was considered in statistical testing when the post-thaw recovery of cryopreserved buds was estimated. The estimated means therefore differed from the actual measured ones, and the confidence intervals of the estimated means were quite wide.

When appropriate preservation methods are selected for germplasm preservation, the cost-effectiveness of the conservation methods is also important criteria [7]. For some fruit trees such as *Malus* spp. and *Diospyros* spp., either the cryoprotocols utilizing in vitro-derived shoot tips or dormant buds can be used [7]. Previously, a cost-benefit analysis of PVS2-vitrification and dormant bud techniques used for cryopreservation of ancient apple cultivars showed that the dormant bud method was most effective in terms of time and labor [52].

We previously reported a procedure for the cryopreservation of blackcurrant cultivars by using the excised shoot tips of in vitro cultured shoots for freezing procedures [41]. Dormant bud cryopreservation can be a time-saving method in genebanking, even if the recovery of buds is done in vitro, because the initiation and the multiplication of in vitro cultures prior to cryopreservation can be omitted. Moreover, a dormant bud protocol may be easier to implement compared to in vitro-based protocols, which may need considerable optimization before they can be applied in practice [39,53,54]. If the recovery of cryopreserved buds is possible by grafting or direct rooting, the whole cryopreservation process can be done without laboratory facilities [9,20]. However, if laboratory facilities are available, the recovery via in vitro offers an opportunity to revive cryopreserved material throughout the year and makes it possible to multiply plantlets via micropropagation [13]. In our experiments, the recovery process in vitro was shown to be highly beneficial: only a small propagule (a shoot tip in the bud) was excised for the initiation of in vitro culture, and when a new shoot started to sprout, it could be excised from the propagule, even if blackening of the basal part of the propagule occurred.

## 4. Materials and Methods

### 4.1. Plant Material

**Figure 3:**
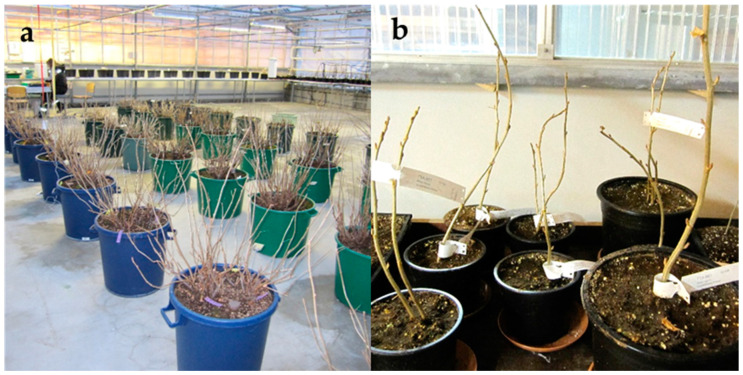
Source plants used for cryopreservation. (**a**) The pre-basic mother plants maintained for plant production. (**b**) Young genebank plants produced for establishment of the new field collection.

In the present study, the dormant buds of a total of 24 blackcurrant cultivars, i.e., 23 listed in Table 2 and cv. Brödtorp, were cryopreserved in 2010–2015 to reinforce the cryobanking of blackcurrant collection. In addition, two black fruited cultivars, Marski and Mikael, and one green fruited cultivar, Vilma, were cryopreserved for a protocol experiment in 2013. In cv. Brödtorp, a larger amount of bud sections compared to the other cultivars were cryopreserved and thawed to perform a rehydration test. All the cryopreserved cultivars, except cv. Hedda, were included in the Finnish national core collection of blackcurrant (*Ribes nigrum* L.). For cryopreservation, dormant buds were collected from the pre-basic mother plants maintained for certified plant production or from genebank plants produced to establish a new field germplasm collection of blackcurrant (Figure 3.). All source plants were maintained in insect-proof greenhouse at the Laukaa in Central Finland (62°19′13″ N, 25°59′36″ E), where the temperature was kept above 4 °C during the winter months. At the time of collection of the buds, the temperature in greenhouse was ranged from 4 to 7 °C, and no bud burst was detected. All source plants were propagated via micropropagation from plants heat-treated to eradicate virus infections. The pre-basic mother plants grown in tubs were tested to be free from pests and diseases regarding to the legislation demands of certified plant production, and they were pruned annually. Genebank plants were tested to be free from blackcurrant reversion virus.

### 4.2. Collection and Handling of Bud Sections

#### 4.2.1. Protocol Experiment

Dormant buds of cultivars Marski, Mikael, and Vilma were collected from greenhouse-maintained pre-basic mother plants used for certified healthy plant production in January 2013. The collected branches were cut into approximately 1.5–2 cm long stem sections, each containing a single bud in the middle of the segment (bud sections). The basal part of stems and the uppermost part of the stems were not used. The length of buds was measured with a calliper. Stem sections containing 3–5 mm long buds were selected for the experiment because they were the most abundant. All bud sections were packed in plastic bags and stored in a cold room at 2 °C for four days.

**Figure 4:**
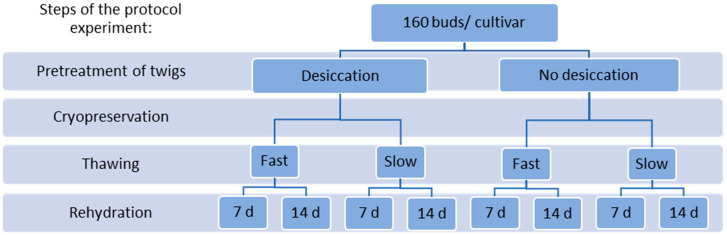
The experimental set-up of the protocol experiment for blackcurrant cvs. Marski, Mikael, and Vilma. Of 160 bud sections of each cultivar, 80 bud section were dehydrated before cryopreservation, and 80 bud sections were cryopreserved without dehydration. Cryopreserved bud sections were thawed either slowly at 2 °C or fast in a water bath at 38 °C, and thereafter rehydrated for 7 days or 14 days. Each treatment combination included 20 bud sections.

**Figure 5:**
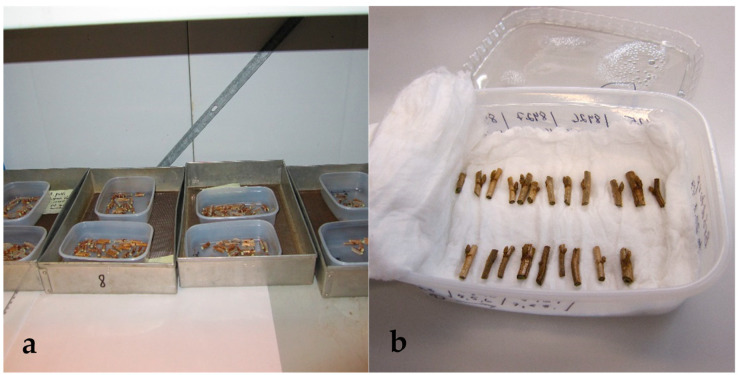
(**a**) Dehydration of bud sections in open freezer containers in cold room at 2 °C. (**b**) Rehydration of bud sections in plastic freezing container in moist cotton wool.

For the protocol experiment, the bud sections of cvs. Mikael, Marski, and Vilma were cryopreserved and thawed according to the experimental design shown in Figure 4. The bud sections were divided into two groups by cultivar, i.e., those to be cryopreserved after desiccation, and those to be cryopreserved without desiccation. Eighty non-desiccated bud sections per cultivar were sealed in cryotubes, i.e., two per one 1.8 mL cryotube (Sarstedt) and placed in cryoboxes (Sarstedt) which were kept in the cold room overnight and cryopreserved the next day. For desiccation, eighty bud sections per cultivar were spread on plastic containers and held unsealed at 2 °C for four days (Figure 5a). The bud sections were then packed in plastic bags for two days before they were sealed in cryotubes and cryopreserved by the same protocol as the non-desiccated bud sections.

To evaluate the moisture content of bud sections prior to cryopreservation, samples of non-desiccated and desiccated bud sections from each pre-basic mother plant were weighed, oven-dried at 82–84 °C for one day and then reweighed. The moisture content of bud sections was determined using the formula (fresh weight − dry weight)/fresh weight × 100 [55]. To evaluate the progress of desiccation, samples of desiccated bud sections were weighed daily, and their moisture content was determined [55].

To test the in vitro culture success of cvs. Marski, Mikael, and Vilma, an additional 10 non-desiccated and 10 desiccated non-cryopreserved buds per cultivar were initiated. The non-desiccated control buds were initiated five days after the buds were collected and then kept in a cold room at 2 °C. Desiccated buds were initiated after 15 days of rehydration. In vitro culture of these control buds was conducted using the same media as described above for cryopreserved buds.

To test the effect of cooling, thawing, and rehydration on the recovery of the non-cryopreserved buds, an additional 24 non-desiccated and 24 desiccated bud sections of cv. Mikael were cooled to −38 °C and thereafter thawed either fast in a water bath or slowly in a cold room. Thawed bud sections (6 per treatment combination) were rehydrated for two weeks or recovered without rehydration.

#### 4.2.2. Cryobanking of Dormant Buds

To perform the cryobanking of blackcurrant collection, dormant buds were collected from a cool greenhouse at January or at the beginning of February, except for the cultivar Ri 289, which was collected and cryopreserved in mid-December. For long-term cryopreservation, only non-desiccated bud sections were cryopreserved. Measured from the sample bud sections of the source plants, the moisture content of bud sections used ranged between 50 and 59%. After cutting the branches, the bud sections were sealed in cryotubes (1 to 3 bud sections per cryotube) and kept at 2 to 4 °C overnight.

### 4.3. Cooling and Cryopreservation of Bud Sections

Pre-cooling and cryopreservation of bud sections was conducted according to the protocol developed for dormant buds of silver birch and aspen [18]. Cryoboxes were placed in the chamber of the programmable freezer (Kryo 10–16 series II with programming unit Kryo 10–22 or Kryo 560-16 with MVR controller, Planer PLC, Sunbury-On-Thames, UK) without lids, and gaps of about 2 cm were left between the boxes using wooden pins. The cryotubes were cooled at 0.17 °C min^−1^ from 0 °C to −38 °C and held at −38 °C for about 30 to 50 min. The cryoboxes were then immersed in liquid nitrogen one by one until the bubbling of liquid nitrogen settled. During immersion, the cryotubes were held in place with a grid. After immersion, the cryoboxes were stored at the gas phase of liquid nitrogen in a cryotank (MVE 1520 Eterne).

### 4.4. Thawing and Rehydration of Bud Sections

In the protocol experiment, bud sections of cvs. Mikael, Marski, and Vilma were thawed either quickly in a water bath or slowly in a cold room. For slow thawing, the bud sections were transferred from the cryotank to a cold room at 2 °C overnight. For fast thawing, the cryovials were placed in a water bath at 37 °C for 3 min. The thawed bud sections were rehydrated in plastic freezing containers inside moist cotton wool at 2–4 °C for 7 or 14 days. Two different rehydration times were used because the results from non-cryopreserved buds of cv. Mikael cooled to −38 °C indicated that the recovery of desiccated buds was very poor without rehydration (data not shown).

To assess the post-cryopreservation viability of 23 cultivars cryopreserved for long term preservation, approximately twenty bud sections per cultivar were thawed after 2 to 42 months of cryostorage. The buds were thawed in a water bath at 37 °C for 3 min and recovered without rehydration. However, a total of 84 bud sections of cv. Brödtorp were thawed to test the necessity of rehydration for the post-cryopreservation recovery of non-desiccated buds. All the buds of cv. Brödtorp were fast-thawed in a water bath, but half the bud sections (42) were rehydrated for 11 days in moist cotton at ca. 4 °C (Figure 5b) before initiation for in vitro cultures, and the remaining 42 buds were initiated without rehydration.

### 4.5. Recovery of Buds In Vitro

The bud sections cryopreserved for the protocol experiment were thawed and recovered in vitro by cultivar so that buds rehydrated for 7 days and 14 days were cultured on the same schedule. The buds of cv. Mikael were thawed after 6 to 7 weeks of cryostorage, the buds of cv. Marski after 16 to 17 weeks, and the buds of cv. Vilma after 31 to 32 weeks of cryostorage. To initiate the in vitro cultures, the rehydrated bud sections were sterilized with 70% ethanol for ca. 20 s, followed by dipping in pure ethanol. The length of the bud was measured on graph paper under the stereomicroscope. The scales and leaves of the bud were removed, and the shoot tip of buds with two- or three-leaf primordia was dissected. The bud type (floral or vegetative), the turgor of primordial leaves (rehydrated or wizened), and the colour of the bud (entirely healthy green or with visible blackening or paleness of leaves, floral primordia, or shoot tip of the bud) were observed and recorded.

**Figure 6:**
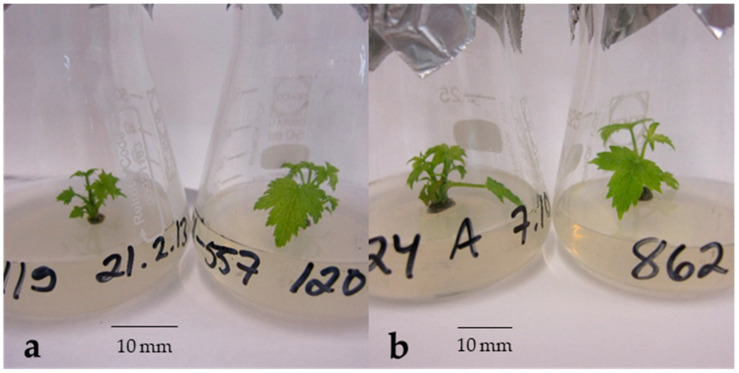
Microplantlets of cv. Vilma after seven weeks in vitro culture in the protocol experiment. (**a**) Shoots regenerated from non-desiccated and non-cryopreserved control buds. (**b**) Shoots regenerated from buds cryopreserved without desiccation and rehydrated 14 day after fast thawing.

The excised propagules were placed in culture tubes containing a WPM [56] culture medium, supplemented as described in Rantala et al. [43]. The culture tubes were transferred to a growth room at 22 °C and kept covered with gauze or foil for 3 days before they were fully exposed to a 16/8 h light/dark photoperiod under two fluorescent tubes (Osram L 36 W/830 Lumilux warm white, Osram, Munich, Germany), with an average photosynthetically activated radiation of 40 to 60 μmol m^−^² s^−^¹. After two weeks, the explants were transferred into Erlenmeyer bottles (25 mL) using G basal medium [57] as described in Rantala et al. [43]. The explants were transferred to a fresh medium after two weeks, and the recovery of the buds was evaluated 7 weeks after the initiation of the in vitro culture (Figure 6). Buds that had produced at least one viable shoot were defined as recovered, and the number and quality of shoots produced by a bud was recorded. In cv. Vilma, a total of 8 buds was rejected from the study because of contaminations, but in the case of cvs. Marski and Mikael, no contaminations were detected.

The buds that were thawed for viability assessments were cultured in vitro using the same culture medias as in the protocol experiment, but the recovery of buds was evaluated 10 weeks after the initiation of cultures.

### 4.6. Statistical Analyses

In the protocol experiment, the recovery percentage of healthy green buds in different categories of treatments or characteristics was studied using contingency tables. The tested effects were pre-treatment (desiccation, no desiccation), thawing method (fast, slow), rehydration (7 days, 14 days), type of bud (flower, vegetative), length of bud (±3 mm, 4–5 mm), and cultivar (Mikael, Marski, Vilma). Fisher’s exact test was used for dichotomous variables, and the Cochran–Mantel–Haenszel (CMH) test for the comparison of the cultivars [58].

The estimated recovery percentages of buds and the number of shoots per regenerated bud after 7 weeks were analysed using the generalised linear mixed model (GLMM). The effects of the cultivar, pre-treatment, thawing method, rehydration, type of bud, size of bud, and all their 2-way and 3-way interactions were used as fixed effects. Statistically non-significant effects were omitted from the final model, and all significant results were reported. Bud sections from the same pre-basic mother plant tub were used as random effects to account for the sampling structure. Binary distribution with logit link was used to analyse the recovery rate.

GLMMs with the assumptions of binary and lognormal distribution were used for the estimated recovery percentage and for the number of regenerated shoots, respectively, for the rehydration experiment with cv. Brödtorp. Time length of rehydration (7 days, 14 days) and type of bud (flower, vegetative) were used as fixed effects. The interaction of these effects was not included in the previous model because there were only six flower buds, and overall, only eight buds did not survive. In terms of the number of regenerated shoots, the length of bud (2‒3 mm or 4‒6 mm) and its interaction with rehydration—but not with the type of bud—were included in the model. In both cases, samples from the same cryovial were used as random effects to account for their possible correlation.

In the viability assessments of cryopreserved cultivars, GLMMs were also used to estimate the recovery of the buds and the number of shoots per regenerated bud. The effects of type of bud (flower, vegetative) and size of bud (small, medium) were added to the models as fixed effects to standardise the comparison of cultivars. Binomial distribution with a logit link and lognormal distribution with an identity link were used to analyse the recovery rate and the number of shoots respectively. Cryovials from the same cryopreserved set were used as random effects.

The estimated means were transformed back to the means of the original scale in all models, but median estimates were used in the case of lognormal distribution. Restricted maximum likelihood (REML) or residual pseudo likelihood (RSPL) estimation methods were used, and the degrees of freedom were calculated using the Kenward–Roger method [59]. Tukey’s method was used for a pairwise comparisons of means [60], with a significance level of α = 0.05. The analyses were performed using the GLIMMIX procedure of the SAS Enterprise Guide 7.15 (SAS Institute Inc., Cary, NC, USA).

## 5. Conclusions

According to our study, the cryopreservation of non-desiccated dormant buds is an applicable method for the long-term preservation of blackcurrant cultivars. Greenhouse-maintained blackcurrant plants are feasible for bud material in their dormant state. The best results were obtained with vegetative buds. The use of young plants should therefore be preferred, or if older plants are used, cultivation practices that keep the plants in their vegetative state should be used before the start of cryopreservation. Recovery via in vitro culture was useful for the regeneration of cryopreserved buds, and visual symptoms of post-cryo damage detected in buds when in vitro cultures were initiated did not predict that the recovery of the buds might fail. The advantage of the introduced protocol for germplasm preservation is that the cryopreservation process takes only a few days. However, in vitro facilities and a programmable freezer, in addition to the cryopreservation devices, are needed. A methodology to distinguish flower buds from vegetative buds in their early stage might further improve the success of the cryopreservation protocol. In addition, variability in the response of different cultivars in recovering and producing shoots in vitro after cryopreservation should always be considered.

## Figures and Tables

**Table 1:** The number of flower buds out of thawed buds and the actual recovery percentages (%) of the cryopreserved buds of blackcurrant cvs. Marski, Mikael, and Vilma per treatment combination. In each treatment combination, 20 bud sections were cryopreserved and thawed either slowly at 2 °C or for 3 min in water bath at 38 °C and rehydrated for 7 or 14 days (d). The actual recovery percentage of buds in vitro was calculated based on the number of regenerated buds related to the number of uncontaminated ones.

		Mikael		Marski		Vilma	
Treatment Combination		Number of Floral Buds	Recovery of Thawed Buds (%)	Number of Floral Buds	Recovery of Thawed Buds (%)	Number of Floral Buds	Recovery of Thawed Buds (%)
Desiccated bud sections							
	Fast thawing + rehydration 7 d	18/20	30	18/20	40	14/19 *	47
	Fast thawing + rehydration 14 d	18/20	30	12/20	80	16/20	60
	Slow thawing + rehydration 7 d	16/20	50	16/20	50	11/20	60
	Slow thawing + rehydration 14 d	18/20	35	17/20	65	12/20	55
Non-desiccated bud sections							
	Fast thawing + rehydration 7 d	18/20	50	16/20	70	18/20	25
	Fast thawing + rehydration 14 d	17/20	50	17/20	80	9/20	70
	Slow thawing + rehydration 7 d	18/20	40	10/20	70	13/20	55
	Slow thawing + rehydration 14 d	18/20	20	17/20	60	10/13 **	46

* 1 bud rejected due to contamination; ** 7 buds rejected due to contamination; d: number of days in rehydration.

**Table 2:** The measured and estimated recovery of buds and measured and estimated number of regenerated shoots per bud of 23 blackcurrant cultivars. The recovery of buds was evaluated after ten weeks of in vitro culture. Results are based on 20 or 21 thawed buds per cultivar, but contaminated initiations were rejected from evaluations. The estimated values take into count the effect of bud type (vegetative or floral) and the size of the bud. CI, 95% confidence intervals.

Cultivar	Actual Recovery of Thawed Buds%	Estimated Recovery of Thawed Buds % (CI)	Actual Number of Regenerated Shoots Per Recovered Bud Mean (CI)	Estimated Number of Regenerated Shoots Per Recovered Bud Mean (CI)
Karila	95	90 (52–99)	8.4 (5.9–10.8)	5.5 (3.9–7.6)
Vilma *	95	90 (52–99)	3.6 (2.9–4.2)	2.7 (1.9–3.7)
Ri 289 *	90	85 (54–96)	5.9 (4.6–7.2)	4.8 (3.5–6.5)
Suvi-7	95	81 (48–95)	7.7 (5.5–9.9)	5.2 (3.8–7.2)
Hedda	75	78 (51–92)	5.1 ((3.9–6.3)	3.9 (2.8–5.5)
Venny *	65	74 (49–90)	2.6 (1.6–3.6)	2.2 (1.6–3.1)
Marski	85	72 (40–90)	4.2 (3.0–5.4)	3.0 (2.1–4.1)
Öjebyn	75	70 (42–88)	3.7 (2.2–5.2)	2.4 (1.7–3.3)
Mortti	80	68 (38–89)	5.1 (4.2–6.0)	3.9 (2.6–5.6)
Vertti *	55	68 (44–85)	3.4 (1.6–5.1)	3.0 (2.0–4.4)
Mikael	70	66 (39–85)	3.7 (3.0–4.5)	2.9 (2.0–4.1)
Ola	70	65 (38–86)	2.1 (1.6–2.6)	1.6 (1.1–2.2)
Pyhtilän Musta	85	63 (26–89)	1.7 (1.2–2.1)	1.1 (0.7–1.6)
Osmola	75	63 (35–84)	5.9 (3.7–8.1)	3.5 (2.5–5.0)
Nikkala	80	60 (29–85)	3.9 (2.3–5.5)	2.4 (1.7–3.5)
Åström	75	59 (32–82)	3.3 (2.0–4.5)	2.1 (1.5–2.9)
Kangosfors	80	59 (29–83)	4.6 (3.1–6.1)	2.9 (2.0–4.1)
Osmolan musta	75	56 (30–78)	5.0 (2.7–2.6)	3.0 (2.1–4.2)
Kuoksan Musta	80	55 (23–84)	2.0 (1.4–2.6)	1.3 (0.9–2.0)
Gerby	70	55 (29–78)	3.0 (2.1–3.9)	2.0 (1.4–2.8)
Matkakoski	65	54 (29–77)	1.5 (1.1–1.9)	1.2 (0.8–1.7)
Lepaan Musta	63	42 (16–73)	3.5 (2.5–4.5)	2.2 (1.6–3.7)
Jänkisjärvi	15	9 (3–29)	1.3 (0–2.8)	1.1 (0.6–2.3)

* Green fruited.

## Data Availability

The data presented in this study will be retained according to the policy of the Natural Resources Institute Finland (Luke) and it will available on request from the corresponding author.
